# Energy Efficient Determinism in WSN through Reverse Packet Elimination

**DOI:** 10.3390/s20102890

**Published:** 2020-05-20

**Authors:** Fredrik Kvist, Andreas Ramstad Urke, Knut Øvsthus

**Affiliations:** 1 Department of Computer Science, Electrical Engineering and Mathematical Sciences, Western Norway University of Applied Sciences, 5063 Bergen, Norway; arur@hvl.no (A.R.U.); kovs@hvl.no (K.Ø.); 2 Department of Information Security and Communication Technology, Norwegian University of Science and Technology, 7491 Trondheim, Norway

**Keywords:** 6TiSCH, packet replication, TSCH, DetNet, WSN, IWSN, IoT

## Abstract

Recently, the industrial wireless sensor network (WSN) has gained attention as a complement to wired networks due to its flexibility and lower installation cost. We present a novel Reverse Packet Elimination (RPE) algorithm implementation at the IPv6 over the TSCH mode of IEEE 802.15.4e (6TiSCH) stack that increases reliability without significantly increasing energy consumption. RPE increases the reliability while conserving energy by transmitting a cancellation packet from the sink towards the sender to reduce unnecessary packets. The evaluation utilized mainly the 6TiSCH Simulator, with additional analytical assessments. We present several evaluation scenarios and compare WSN with and without RPE. In a WSN where each link had a packet reception rate of 70%, RPE increased the reliability with 11.8%. Furthermore, the average latency decreased with 39.1%. The average energy consumption increased with 19.8% when utilizing RPE. However, the network lifetime, i.e., the time before the first node experiences battery depletion increases slightly, which is a significant improvement compared to alternative replication mechanisms.

## 1. Introduction

Internet of Things (IoT) is emerging with an expectation of billions of connected devices to the Internet. These devices utilize low-power wireless communication technologies enabling long battery life and reliable end-to-end transmissions [1]. Capitalizing on this development, industries shift towards the Industrial Internet of Things (IIoT) through concepts such as Wireless Sensor Networks (WSN). Utilizing WSN decreases installation cost as compared to infrastructures for wired sensor networks [2]. According to [3], the cost of installing cables across an industrial plant can run from USD 100s/ft to USD 1000s/ft.

In 2016 the Institute of Electrical and Electronics Engineers (IEEE) 802.15.4 low-rate wireless standard [4] was amended with a new Time-Slotted Channel Hopping (TSCH) media access control targeting industrial applications. The IPv6 over the TSCH mode of IEEE 802.15.4e (6TiSCH) working group [5] was started by Internet Engineering Task Force (IETF) with an intent to enable IP traffic for IIoT utilizing TSCH. In addition, 6TiSCH follows IETF Deterministic Network (DetNet) working group architecture [6], aiming to standardize layer three traffic in terms of networks requiring deterministic capabilities. Wireless networks have a higher probability of packet loss compared to wired due to interference and channel disturbance. Hence, 6TiSCH recommends packet replication in its architecture in order to increase the reliability [5]. However, in WSN packet replication causes higher energy consumption [7] and, thus, reduced network lifetime. Industrial networks face tough lifetime requirements that WSN struggles to meet with packet replication.

This paper proposes and investigates a novel technique utilizing a Reverse Packet Elimination (RPE) algorithm. Packet replication is used to increase the reliability of the network. In addition, RPE limits the negative effect of reduced network lifetime as the replicated packets are cancelled when the original packet successfully reached its destination. This is achieved as the RPE enables the sink to send a packet down the alternative path to eliminate the replicated packet from being transmitted upstream. The energy consumption is reduced as a RPE packet is smaller than an upstream packet, and hence utilizing less resources to traverse the path. In addition, this paper examines the effects of introducing an adjustable replication delay, where the transmission of the replicated packet is delayed. The motivation for this is to avoid transmitting the replication packet if the original packet has arrived at its destination (the sink).

## 2. Background

Assume a scenario where an Industrial WSN (IWSN) establishes communication in a feedback loop that compensates for a control variable in a system. The system defines the update time that the control loop must fulfill in order to keep the system stable. Hence, a WSN must ensure that the control variable is delivered within a minimum latency according to these requirements.

Replicating packets is a technique to ensure higher reliability. This means that each packet is duplicated and sent along two disjoint paths. At the destination, only the first packet is kept, and the copied packets are discarded as they arrive. Packet replication creates redundant data traffic, in order to avoid a single point of failure. In short, this increases reliability with the drawback of increased traffic and energy consumption as more packets traverse the network.

### 2.1. DetNet

DetNet working group [8], intend to provide an architecture for networks requiring guaranteed bandwidth, extremely low loss, and an upper bound on maximum end-to-end latency at layer 3 paths across multiple layer 2 networks. The DetNet working group collaborates with the IEEE 802.1 Time-Sensitive Networking task group [9] which is improving on layer 2 operations to ensure deterministic capabilities. In essence, DetNet and TSN define a common architecture for both Layer 2 and Layer 3 to ensure applications requiring determinism can work across both layers.

### 2.2. TSCH

**Figure 1:**
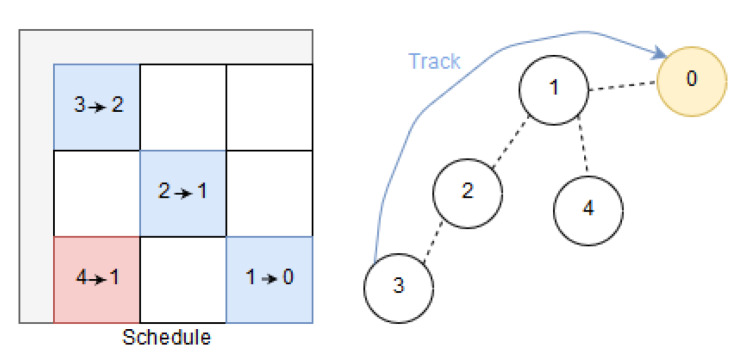
Illustration of a IPv6 over the TSCH mode of IEEE 802.15.4e (6TiSCH) track from 3 to 0.

Time-Slotted Channel Hopping (TSCH) media access divides time into timeslots and assigns them in order to avoid contention. In addition, channel hopping combats link failures caused by external interference and multi-path fading. Together, this enables TSCH to achieve high reliability. Furthermore, TSCH opens for low-power operation as a device may sleep if the schedule of slots does not indicate otherwise. The length of a timeslot is typically 10 ms and timeslots are grouped into repeating slotframes commonly consisting of 101 timeslots [10]. A single element in the TSCH schedule is known as a cell. A cell in the slotframe is identified by a timeslot- and channel-offset [11]. It allows for one transmission opportunity with an optional acknowledgment. The allocation of cells in the slotframe is typically referred to as the schedule. Figure 1 shows a slotframe which is three timeslots long where four cells have been scheduled. Cells can be organized in what the 6TiSCH architecture denote as tracks [5] (which can also be part of a DetNet flow). These are in principle reserved resources between two entities, e.g., a node and a sink. In essence, cells are reserved for a specific track, and only traffic associated with this track may utilize the cells. Multiple cells bundled together increase the probability of successful transmission within a slotframe. Tracks are shaped as a Directed Acyclic Graph (DAG), and support multi-path forwarding and route around failures [5]. An illustration of a track is provided in Figure 1.

### 2.3. 6TiSCH

6TiSCH working group [5] combines standards developed from both IETF and IEEE, i.e., the industry-enabling TSCH MAC, with an Internet-enabling IPv6-based upper layer. This include amongst other the Routing Protocol for Low-Power and Lossy Networks (RPL) [12] and UDP. Altogether this constitutes the 6TiSCH stack which aims for end-to-end connectivity with deterministic capabilities, low power operation and robustness [3]. It is envisioned to enable the Industrial Internet of Things (IIoT), and includes applications such as industrial process monitoring and control. A comprehensive description and tutorial on 6TiSCH can be found in [13].

### 2.4. RAW

The recently formed Reliable and Available Wireless (RAW) working group [14] at IETF extends the work at DetNet into new use-cases for wireless applications in aeronautics, robotics, industry, gaming, etc. While DetNet mainly focus on wired connections, RAW addresses wireless-specific topics for a move towards deterministic properties over technologies such as 5G and 6TiSCH. RAW is applied at the forwarding-plane at layer 3, i.e., the end-to-end routing decision is still taken at the controller-plane by, e.g., a centralized DetNet Path Computation Element (PCE). Yet the per-packet forwarding decision is handled by RAW with an aim to ensure the end-to-end path has high reliability and availability., e.g., if an intermediary node fails or a link is degraded, RAW offer mechanisms such as packet retransmission, replication, elimination, and overhearing (PAREO) to mitigate the failure [15].

## 3. Related Work

Our work relates to the effort of increasing the robustness of WSN conducted by The Leapfrog Collaboration (LFC) [16,17]. This work was recently extended in [18] which proposes PAREO where packet retransmission, replication, and overhearing is evaluated in combination. However, to the best of our knowledge, no related work exists in terms of a reverse mechanism or replication delay. Although, in [19] the authors visit the idea of a reverse packet mechanism in Ethernet, with the reversing of packet elimination to free bandwidth upstream. However, the proposal is not further discussed or investigated.

Packet replication and elimination techniques in WSN increases energy consumption significantly. Such a rise in energy consumption is expected as the number of packets traversing the network at minimum doubles. This is shown in [7], where the authors implement disjoint paths and transmit a replicated packet at each path. In short, the source duplicates the packet and the sink drops the last arriving packet. The authors show that packet replication decreased packets lost with almost 90%, however, the energy consumption in their scenario increased with 86.3%.

Leapfrog Collaboration (LFC) utilizes the same principle as [7] by dropping the last arriving packet. However, LFC implements replication and dropping at each hop. By taking advantage of promiscuous overhearing, LFC utilizes a Leapfrog Beacon which enables all nodes to make the same cell available for receiving (RX). Opening the same cell allows all nodes except the node transmitting to listen for data, and LFC uses the overhearing function to double the probability that the packet will traverse over the DODAG network. As a result, delay decreases significantly as the packet propagates the favorable path. If no nodes are within sensing range, normal retransmission schemes are used. LFC achieves minimum end-to-end reliability of 99.1% for all simulations, with the lowest packet delivery ratio (PDR) link quality at 70% [17]. However, with a drawback as the energy consumption increases. LFC reduces the average delay by 41% compared to schemes with four retransmission attempts [17].

## 4. Reverse Packet Elimination

This paper proposes a novel Reverse Packet Elimination (RPE) algorithm that aims at increasing reliability while limiting the negative effect of reduced network lifetime. Similar to [7] two disjoint paths are created from the source to the sink. The motivation for using packet replication is to increase the reliability of the WSN. It is assumed, as in [7], that the scheduler establishes the paths or tracks based on routing and connectivity information obtained through the control plane links [20]. The disjoint paths or tracks may be computed by a centralized PCE running, e.g., the Traffic Aware Scheduling Algorithm (TASA) as proposed by [21]. The source node replicates its data packet, that is one candidate packet for each path. However, the node transmits initially only one of the packets and detains the second. The following description discusses the motivation and criterion for delaying the second packets. Forwarding two packets increases the traffic load and thus the energy usage in the network. Furthermore, as the packets have the same size, they have the same drop probability over lossy links (links with high Bit Error Rate (BER)). In addition, adding traffic increases the funneling effect. The first packet that arrives at the sink, triggers the sink to send a small RPE-cancelling packet down the alternative path from the path of the packet.

**Figure 2:**
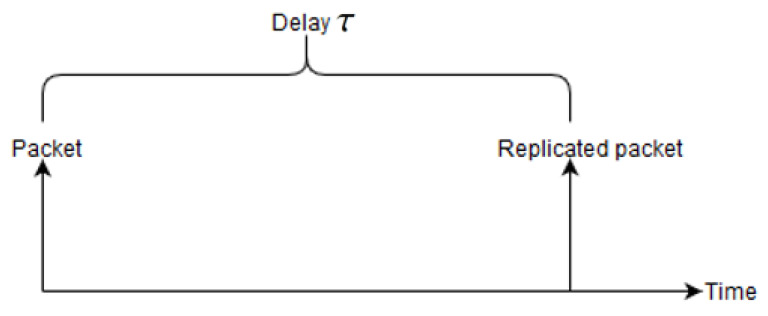
Illustration of how the replicated is withheld by τ.

The RPE-cancelling packet searches for the copy that is propagating upstream and cancels it, i.e., stop further forwarding of the packet in order to conserve energy. The basic structure of the RPE algorithm is packet replication and enabling the sink to cancel the second packet. However, a second feature is introduced, this is a delay of transmitting the second packet. Depending on the requirements for delay and robustness, the second copy can be withheld by an adjustable time delay τ. This is depicted in Figure 2.

**Figure 3:**
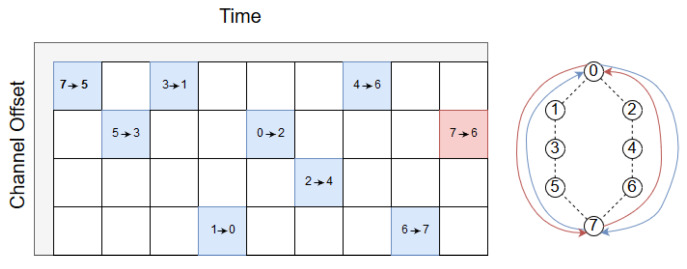
Example of a Path Computation Element (PCE) scheduling tracks from source to sink.

**Figure 4:**
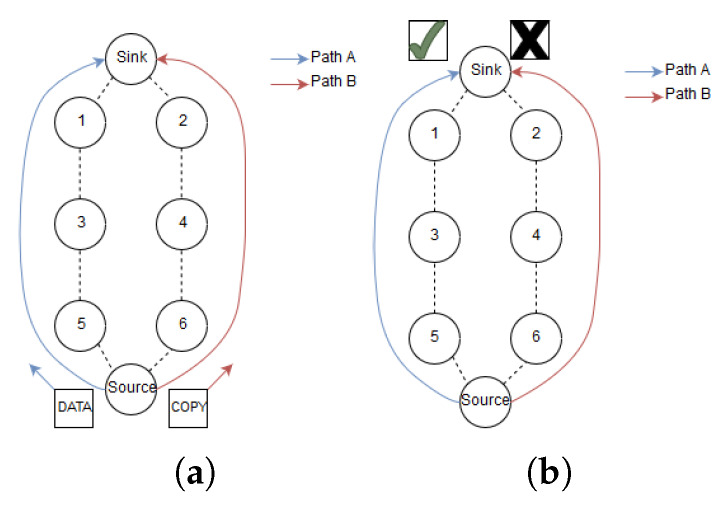
Illustration of Revere Packet Elimination (RPE) step 1 (**a**) and step 2 (**b**).

**Figure 5:**
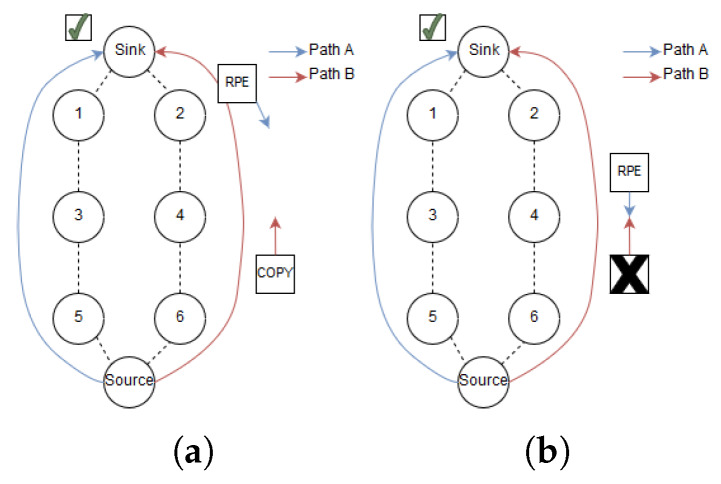
Illustration of RPE step 3 (**a**) and step 4 (**b**).

An example network illustrating RPE is shown at the right hand side of Figure 3. The source node is node number 7 and the sink is node number 0. From the source to the sink two disjoint paths are shown, as Path A and Path B. The source node holds initially two copies of the packet (the original and the replicated packet). Our proposed RPE method is described in six steps:

1. A PCE schedules 6TiSCH tracks in daisy chains with two disjoint paths, Path A and Path B. Path A is by design scheduled to send the first packet. As shown in Figure 3, blue cells make up a track from source to sink (0).

2. The source node replicates the data packet and sends it along Path A. Next, the sender schedules the copied packet to be transmitted along path B, which is disjoint from A. Both paths have explicit routes towards the sink as seen in Figure 4a. The copied packet along Path B is delayed dependent on the upper bound latency of the application. The motivation for delaying this packet will be discussed further in the following text.

3. The two packets propagate along the two disjoint paths towards the sink. As depicted in Figure 4b, the packet arriving last is discarded if both packets arrive at the sink before the sink could send an RPE-cancelling packet.

4. If they do not arrive simultaneously, the sink sends an RPE-cancelling packet down the alternative path from path of the arriving packet as shown in Figure 5a. For example, if the Path A packet arrives first, the RPE-cancelling packet is sent down Path B and vice versa.

5. The RPE-cancelling packet traverses towards the source. In each visiting node, the packet triggers a search in the node’s queues for a copy of the packet that arrived at the sink. As illustrated in Figure 5b. If a packet is located with an identical sequence number, this packet is dropped.

6. If an RPE-cancelling packet arrives at the source (looping back) before the replicated packet is sent, the source revokes the transmission of the copied packet.

### 4.1. Packet Size

In our investigations, we utilize a max packet size of 127 bytes for data packets and the RPE-cancelling packets are set to 23 bytes. 23 bytes is the minimum packet size of an 802.15.4 frame carrying the necessary headers and two bytes containing the RPE-cancelling payload.

Link quality is defined by its BER and the BER differs for each link according to its propagation characteristics. Assuming the BER of each bit is independent, if there is a bit error in a packet, the packet is dropped. PER in Equation (1) denotes the packet error rate for a packet with n bits.

(1) $$PER=1-{\left(1-P_{b}\right)}^{n_{bits}}$$

Writing packet reception rate (PRR) as PRR = 1 − PER, the following relation is found between the PRR of two packets with different sizes (short and long packets, namely $PRR_{s}$ and $PRR_{l}$), with both packets experience the same BER.

(2) $$PRR_{s}=1-PRR_{l}^{\frac{L_{s}}{L_{l}}}$$

where $L_{s}$ and $L_{l}$ are the numbers of bits in the short packet and long packet respectively. Equation (2) shows that the packet size matters in terms of PRR. Since the cancelling packet is significantly smaller than the larger upstream packet it has some advantages; it has higher PRR. Furthermore, the smaller RPE-cancelling packets require less transmission time as they contain fewer bits than a large data packet. This causes reduced energy consumption. Finally, due to higher PRR, the time the radio is active is lower, energy consumption decreases due to fewer retransmission attempts. From Equation (2) we find that if a 127 bytes data packet experiences a PRR = 70%, then a 23 bytes RPE-cancelling packet experiences a PRR = 93.7% under the same BER condition.

To put this into perspective, assume the topology in Figure 4 where the source sends replicated packets along Path A and Path B. After a while, the link quality between 1 and 3 deteriorates from 70% to 20% for the 127 bytes packet. Assume that a 127 bytes packet then has a PRR=20%, a 23 bytes RPE-cancelling packet have PRR=74.7% at the same link. This means that theoretically, a RPE-cancelling packet can traverse from 1 to 3 and stop the large packet from utilizing all its retransmission attempts. In essence, this decreases resource utilization at nodes when a change in link quality occurs. As mentioned earlier, smaller packets consume less energy than larger packets. The main contributor to energy consumption in WSN is transmitting and receiving packets [22]. Based on this understanding the energy consumption of transmitting and receiving a packet is proportional to the packet size. Thus, transmitting short RPE-cancelling packets requires less energy than longer data packets.

### 4.2. Selecting Delays

This section discusses delaying the replicated data packet. Clearly, this depends on the requirements imposed on the network by the system. For example, a WSN in a control loop must supply the controller with data within a time frame in order to keep the process stable. Assume the maximum permitted delay imposed on the network is $T_{crit}$, where we have reduced the number to safeguard the system. The maximum delay $T_{crit}$ is a design criterion for the delay between the source and the sink, $T_{onepath}$. If the delay from source to sink meets the design criteria ($T_{crit}$), but with limited margin, the advantage of RPE is as described above. However, if there is a margin, for example, that the delay between source and sink permits that the replicated packet is delayed a time, τ, before it is transmitted then the second advantage of RPE comes to play. RPE provides energy savings and preserves the robustness when the replicated packet can be delayed a time τ before it is transmitted. This means that if the first packet is lost, the replicated packet meets the deadline as it reaches the sink. In order to reduce the traffic, RPE enables the sink to cancel unnecessary transmission of the replication packets by sending RPE-cancelling packets. As pointed out earlier, this reduces the energy consumption in the WSN. In addition, it reduces the funneling effect caused by added data traffic imposed on the nodes close to the sink (the topic is presented in the following text). Next, we discuss the time delay, τ.

Selected replication delays τ are as follows: 1, 8, 816, 1624. As mentioned earlier τ is always given in slots, and it is the delay from the time the first copy was sent. Our assessment of delaying the replication packet is based on three scenarios. Firstly, we assess the situation where both packets are transmitted without waiting for the cancelling packet. Secondly, we assess the situation where the delay is larger than the round-trip time from the source to the sink. This means that the replication packet is delayed so much that the first packet reaches the sink and the cancelling packet reaches back to the source. In our example, this equals eight slots. However, if the first packet experiences delay caused by retransmission, the second packet is transmitted. Delaying the replication packet further takes retransmission into account. Thirdly, we assess the situation where the replication packet is delayed to the extent that both the path A packet and the RPE-cancelling packet have exhausted all their retransmission attempts.

**Figure 6:**
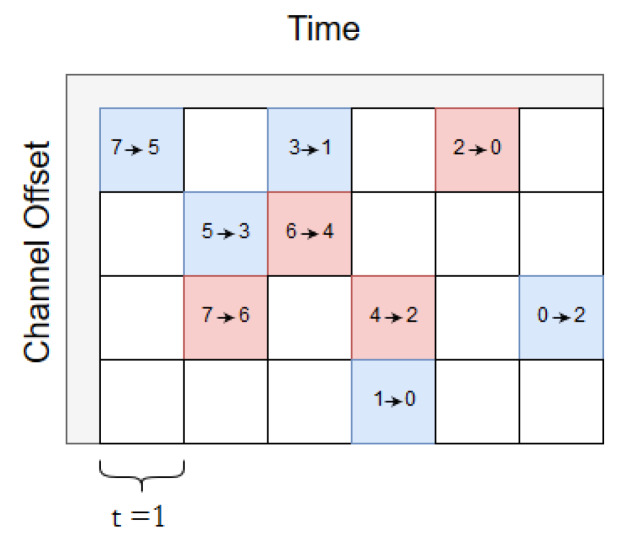
Scheduling a delay of τ=1.

When τ=1 slot, the source always sends two copies. This delay has the lowest latency (for both packets) since both packets have the shortest scheduled delay. Moreover, both packets traverse Path A and Path B towards the sink with a time difference of a slot as illustrated in Figure 6. As can be seen in the figure, the RPE-cancelling packet, from the sink towards the source, must wait for one slot. The reason is that according to the scheduler, the sink must first listen for incoming packets in the 2→0-cell (Path B). If the replicated packet did not arrive, the next cell, marked as 0→2, is allocated to the RPE-cancelling packet. Clearly, switching these two cells is an option, saving one transmission from node 2, at the expense of increasing the delay along path B by one slot.

**Figure 7:**
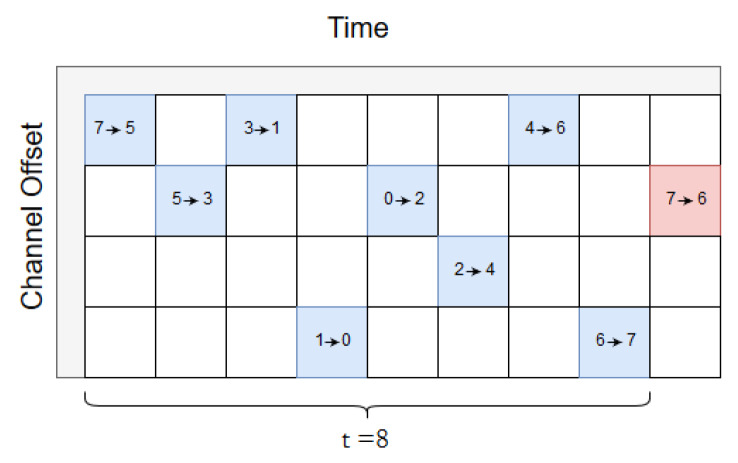
Scheduling a delay of τ=8.

The next delay is τ = 8, Figure 7 depicts the scheduler. τ = 8 slots are selected based on the number of hops between source and sink, since there are 4 hops to sink and 4 hops in return to the source. As can be seen, the Path B packet will not be sent if the Path A packet arrives successfully according to the scheduler, and the reverse path back to the source do likewise. When τ = 8 slots, the Path A packet has enough time to reach the sink and trigger a message to stop the source from transmitting the replicated packet.

If τ>101, the replicated packet is scheduled with a delay of at least one slotframe later. A delay of τ = 1624 slots is the maximum latency of the packet looping back to the source in this topology. The delay equals 16 slotframes. In essence, the Path A packet is given the time to utilize all transmission attempts before the Path B packet is transmitted. A transmission fails if a packet was lost or its acknowledgement (ACK) fails during transmission. Hence, nodes have several attempts to ensure successful transmission. However, if a transmission fails in the current configuration, a node has to wait until the next slotframe iteration before trying again. Finally, τ = 824 slots was selected to investigate the difference between the short and the long delays. With τ = 824 slots, the delay is cut from 16 slotframes to eight slotframes. The delays are topology specific except τ = 1 slot.

### 4.3. Path Considerations

RPE assumes a routing protocol capable of providing upward routes for data and replicated data, as well as downward routes for RPE-cancelling packets. The RPL routing protocol which is part of the 6TiSCH suite offers both these capabilities. Since the downward RPE-cancelling packet always originates at the sink node, both storing and non-storing RPL mode may be utilized with RPE.

The RPL preferred parent selection needs to be adapted such that the source node can employ both Path A and Path B to forward traffic. Such a mechanism is implemented in [18]. Selection of different parents, namely Preferred Parent and Alternative Parent, is in itself a field to be studied, and is not treated further here. An example of work in this direction can be found [23].

RPE requires routing and accompanying schedules to yield disjoint paths through the network. Investigation into this assumption, such as, e.g., the requirement on network density, is deferred to future work. As described above, for our evaluation we assume a centralized PCE as envisioned in, e.g., 6TiSCH to provide this capability. This centralized strategy seems to be an intuitive solution for these cases since it has an end-to-end awareness such that disjoint paths can be guaranteed. The 6TiSCH hop-by-hop scheduling model [5] may also be feasible since it has the same end-to-end property.

## 5. Simulation Setup

This section investigates our proposed Reverse Packet Elimination method. It was implemented in the 6TiSCH simulator [24]. The network topology selected was influenced by Leapfrog Collaboration [16,17], and the configuration contains 8 nodes. As seen in Figure 4, node 0 is the sink and node 7 is the source. The network is configured to utilize the full implementation of 6TiSCH available in the simulator. The parameters utilized in the simulation are listed in Table 1.

### 5.1. Scenarios

Several different configurations were simulated to assess the effects of the Reverse Packet Elimination technique. These configurations are listed here:

#### 5.1.1. Single Path

Is a scenario where traffic is only sent from the source to sink through Path A. At Path B, no data traffic occurs, only normal 6TiSCH generated traffic traverses along the path. This scenario defines normal 6TiSCH operation without packet replication or packet elimination. In the following text and in the figures, the Single Path scenario is referred to as SP.

#### 5.1.2. Dual Path

Dual Path scenario depends on the existence of two disjoint paths between the source and the sink, which means that the two paths do not share any relaying nodes. One path is utilized by the original packet, while a replicated packet is sent up the second path. In addition, there is no RPE-packet generated at the sink. Dual Path is a simple implementation of the Packet Replication, Elimination, and Ordering Functions (PREOF) as described in DetNet Architecture [6]. The following text refers to this scenario as DP.

#### 5.1.3. RPE

RPE refers to the implementation of our novel Reverse Packet Elimination proposal. This scenario is listed with corresponding sending delay, τ, in the text and the figures. The delay is given in number of TSCH slots. In all RPE scenarios, the traffic is sent at Path A with the replicated copies being sent at Path B (if required).

#### 5.1.4. RPE Overprovisioning

In this scenario the PCE schedules an extra transmitting (TX) and RX slot for each upstream hop. Otherwise, this refers to RPE operation with τ = 8 slots. In the figures, RPE Overprovisioning is referred to as OP.

## 6. Performance Results

**Figure 8:**
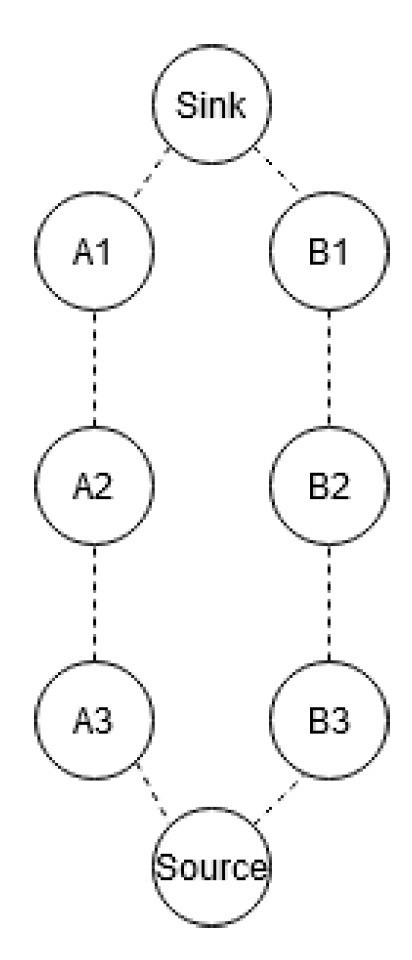
Nodes with corresponding path and hop count.

Our hypothesis is that RPE increases the WSN’s reliability without substantial decreased network lifetime. Presumably, RPE can only support system where its critical time delay compared to the WSN round trip time (from source to sink) meets the requirements presented here. This section assesses the performance of RPE in light of these challenges. As seen in Figure 8, nodes are named according to their path and hop count, such as A2 or B1.

### 6.1. Reliability

**Figure 9:**
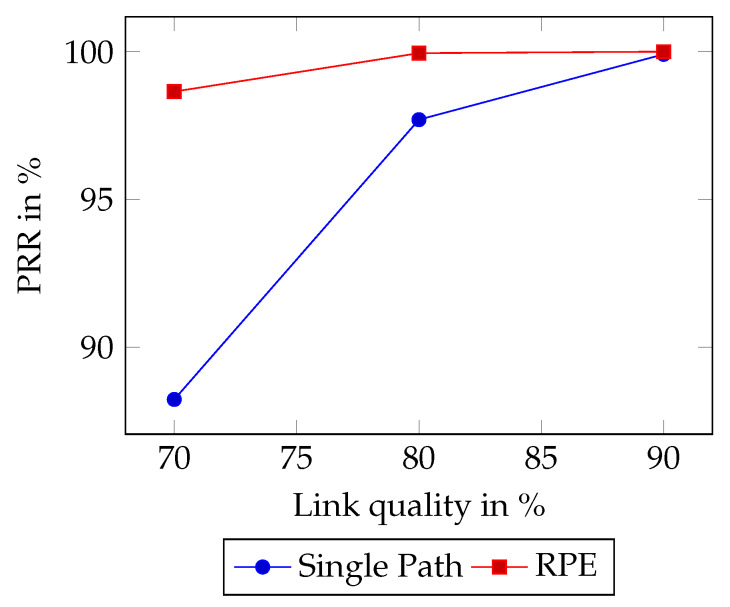
Packet reception ratios for Single Path and RPE.

The RPE method is investigated for a WSN with links that experience packet drops. Following the same investigations as LFC [16], we assess links with PRR 70% increasing in increments of 10% until a 100% PRR. RPE, with τ = 8 slots, is compared with the Single Path scenario. It was found that all the scenarios involving packet replication showed a negligible difference in reliability as from the sinks point of view the delay τ does not affect the PRR. Hence, in Figure 9 SP is only compared to RPE τ=8 for simplicity.

Figure 9 shows that RPE increases reliability compared to Single Path. Single Path was found to have a reliability of 88.24% when the link quality was 70%. At the same link quality, RPE increases the reliability to 98.65%. Moreover, the reliability of WSN with links with link quality of 80% shows significant improvements as Single Path had a packet reception ratio of 97.7% compared to RPE with a success rate of 99.95%. Lastly, at 90% link quality, the gap in reliability shrinks to a difference of 0.09% as RPE delivers a 100.0% delivery ratio. However, there is packet loss involved, and over time statistically, a packet will be dropped.

### 6.2. Distribution of RPE-Cancelling Packets

**Figure 10:**
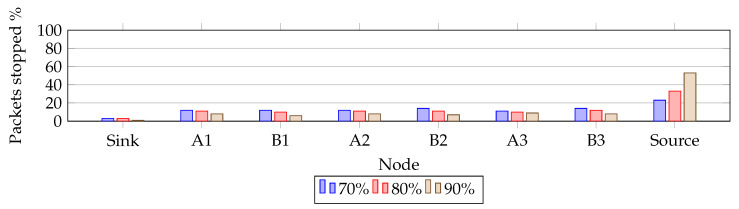
Packet elimination distribution for τ=8.

**Figure 11:**
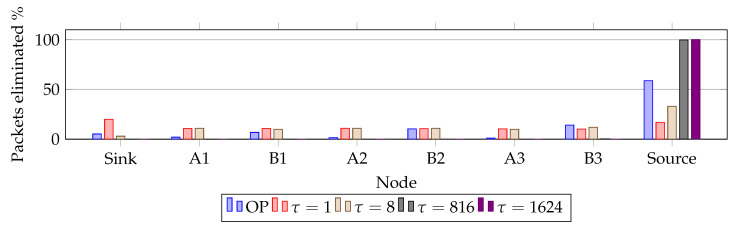
Packet elimination distribution with 80% link quality.

Clearly, our RPE method creates additional traffic in the network compared to a single path. In order to investigate the negative effects of increased traffic, we investigated where the packets are eliminated. Figure 10 and Figure 11 presents the findings. They show where, along the paths, the RPE packets are eliminated. Figure 10 shows how different PRR values change the elimination location. The reduced link quality causes the elimination to spread across all nodes.

Figure 11 shows at which node the replicated packet is eliminated when the link quality is 80%. In the figure various RPE delays are compared with RPE Overprovisioning (OP). It shows how τ = 816 and τ = 1624 yielded similar results. However, τ = 816 experiences that a few packets were dropped along Path B, but over 30 runs the average value recorded was less than 0.1%. Although, at node B3 an average of 4 packets were eliminated accounting for 0.24% of the drops. Summarizing, τ = 816 and τ = 1624 shows 99.76% and 100% packet elimination occur at the sink. Delay effects are discussed in more detail in the following text.

### 6.3. Latencies

This section presents the simulation results with regards to latency in data sent from the source to the sink. To provide a baseline, Table 2 shows how the maximum latency increase with longer replication delays and minimum theoretical increase when overprovisioning.

The simulated latencies show the average, minimum, maximum, and the 99th percentile. Maximum latency presents the highest value recorded for all runs. What is more, maximum latencies listen in Table 3 equals theoretical maximum latencies.

The latencies of the 99th percentile are presented in Table 4. Single Path has a higher 99th percentile for all link qualities compared to RPE with τ = 1 and τ = 8. At PRR = 90% the difference is 1.89 s between RPE with τ = 1 and Single Path. Increasing τ from one slot to eight slots increases 99th percentile latency with 40 ms. This is interesting as the difference in sending the replicated packet is increased from 10 ms to eight times as high at 80 ms, but 99th percentile latency is only increased with 40 ms. Decreasing the link quality increases the 99th percentile latencies for all scenarios. What is more, the difference between τ = 1 and τ = 8 is fairly similar for each value of PRR (70%, 80%, and 90%).

### 6.4. Node Lifetime

The network lifetime is an important parameter; it has, however, several definitions [25]. Here we adopt the definition that the network lifetime equals the time until the energy of the first node depletes. In our simulations, we have utilized a battery size of 2821.5 mAh.

**Figure 12:**
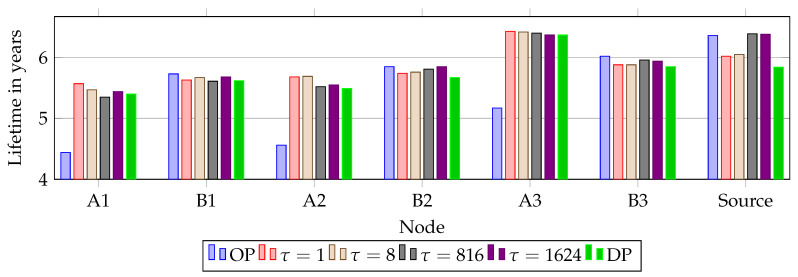
Scenarios and their average node lifetimes in years (Note: y-axis starts at 4 years).

Figure 12 presents the average node lifetimes in years for all simulations. Due to the extra TX/RX cell when Overprovisioning (OP), Path A has a significantly lower battery lifetime compared to Path B. Comparing Path A of OP with alternative RPE shows that OP has the lowest lifetime. It shows an imbalance as Path A is over-utilized and Path B not utilized. Dual Path (DP) also has worse performance at each node, compared to all simulations using RPE.

All simulations show a correlation between lifetime and where the upstream copy was eliminated. τ = 816, τ = 1624 and OP has the highest number of packets eliminated at the source. Hence, the source has the highest lifetime, as it does not send the replicated packet as often as for the other scenarios. It is very interesting to note the decreased load on node A1 when τ = 1. This is due to the frequency of alternating drops at sink, A1 and B1. On the other hand, it causes the source to have the lowest battery life of all RPE scenarios. The reason for the lowest battery life at the source is as mentioned in the selection of delays, that the source always sends both copies.

**Figure 13:**
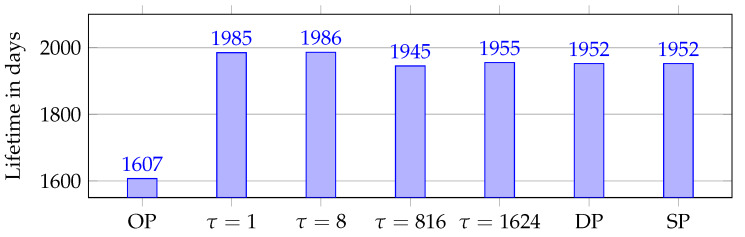
Lowest node lifetime for the different scenarios.

We found that for all simulations the first node to run out of power is A1, except in τ = 1 and τ = 8 were in approximately $\frac{1}{30}$ simulations B1 had the shortest lifetime. Figure 13 presents the lowest node lifetimes. As seen previously in Figure 11, no elimination was registered when using τ = 816 or τ = 1624, causing traffic to behave as DP and SP. This is reflected in the lowest lifetime as for both scenarios it is within 7 days of SP and DP. The effects of OP are clear with A1 running out of battery 338 days before any other version due to the over-utilization at Path A. As mentioned in Section 4.1, packet size matters, and the effects of utilizing RPE is clear on the node lifetimes.

### 6.5. Funneling Effect

**Figure 14:**
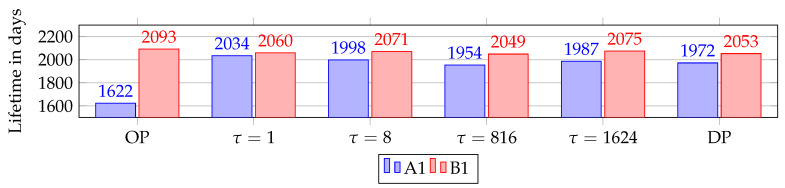
Funneling effect at A1 and B1. Represented with node lifetime in days. (Note: y-axis starts at 1500 days).

The funneling effect occurs as the nodes closer to the sink have a higher load than nodes further away, due to the concentration of traffic on fewer nodes. This is shown in Figure 14 where the average lifetime at A1 and B1 for all versions using packet replication are listed. The short delay, τ = 1, return the evenest distribution between A1 and B1 since packet elimination occurs at a higher frequency at these nodes as seen in Figure 14. In addition, τ = 8 decreases the funneling effect as well, but not as much as τ = 1. On the other hand, increasing replication delay τ decreases the load on Path B. Moreover, overprovisioning has the lowest latency, resulting in the highest load on Path A, and lowest load on Path B. As previously mentioned, this is due to the over-utilization on Path A. The funneling effect can be seen for all nodes in Figure 14 where lifetime increases with distance from the sink.

### 6.6. Average Energy Consumption

**Figure 15:**
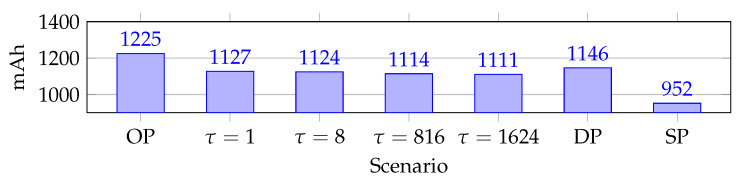
Average current consumption.

Energy consumption is critical in industrial WSN. As stated in the introduction, our hypothesis is that RPE reduces energy consumption. The energy consumption is found from the current consumption, since the voltage is constant. Different WSN designs produce different average current consumption as topology influences energy consumption. The average accumulated current consumption is shown in Figure 15. The effects of packet replication are clear, as shown in Figure 15, Single Path utilizes 159 mAh less than second-place τ = 1624. This illustrates the energy cost of replication in general.

The effects of overprovisioning are seen with the highest accumulated current consumption at 1225 mAh. This increase is due to the extra cells, which causes more idle listening than the other scenarios.

Another key point is that the RPE scenarios have a lower average current consumption compared to Dual Path. A decrease in average current consumption is important as this topology does not represent a complete network. In fact, real network data traverse from other paths as well. Hence, decreased current consumption decreases due to reduced load on certain nodes due to funneling effects.

## 7. Discussions

Our analysis follows the methods used by LFC, and as LFC we investigate only a section of a WSN. A complete WSN with several paths puts requirements on the network design as two paths must be available for critical traffic flows. Our RPE method imposes only the requirement that the two paths are disjoint. Paths may hold a different number of nodes; this difference will alter the performance in terms of finding the optimal delay of the replication packet. Clearly, the method depends on the requirements imposed on the WSN in terms of delay tolerances and the performance of the WSN. Our results show optimal delay equals the time delay from the source to the sink and back along the second path. In our network, this was a delay of eight slots. The reason is that in an environment with no packet losses, the tracks allow a packet to loopback and stop the source from sending the replicated packet. Based on the results with 80% link quality, τ = 8 achieves the best traits of all replication delays. Comparing our RPE with scenarios Dual Path and Single Path with PRR = 80%, the lowest node lifetime increased with 1.74%. Next, the average latency is reduced with 39.1% and reliability increases from 97.7% to 99.95%, when comparing our RPE with Single Path. A natural disadvantage with the RPE proposal is the increase in average current consumption compared to using a Single Path. This is due to extra tracks and packets traversing disjoint paths. However, packet replication is recommended by DetNet architecture and RPE decreases the average current consumption compared to Dual Path, increases with 18% compared to Single Path. Finally, if low average latency is critical, it is recommended to utilize Overprovisioning. Overprovisioning has a 71.2% lower average latency than τ=8. However, this comes with an 18.34% reduction in the lowest node lifetime.

## 8. Conclusions

A novel method, Reverse Packet Elimination (RPE), for packet replication is proposed and analyzed in 6TiSCH. RPE delays the replicated packet from being transmitted and implements a method for cancelling the transmission of the replication packet if the original packet reached its destination. RPE enables WSN with higher reliability, lower average latency and increased node lifetime compared to traditional WSN. Clearly, as with any packet replication method the traffic increases and thus the average energy consumption in the network increases. However, the method of cancelling the replication packet proves effective as it reduces unnecessary transmissions. Related work Leapfrog Collaboration (LFC) finds a 99.1% worst case reliability, with the disadvantage of increasing energy consumption. Our method has similar reliability as LFC, as we got worst-case reliability of 97.7%. Another key point, RPE decreases the funneling effect, thus reducing the energy consumption of nodes closer to the sink and increasing the WSN lifetime. In fact, the lowest node lifetime increased by 1.74% compared to not utilizing any packet replication. However, a drawback of RPE versus LFC is a slight increase in average latency due to the delayed replication packet. This implies that RPE is not optimized for WSN with the most extreme latency requirements. LFC reports a 41% reduction in average latency compared to standard retransmission schemes, while we found that RPE has a 39.1% reduction with equal retransmission attempts. Moreover, LFC is by design dependent on high node density. RPE requires only two disjoint paths to the sink. This makes RPE less dependable on topology and presumably suitable for other WSNs.

## 9. Future Work

The proposed RPE method uses dedicated RPE-cancelling packets for signaling the successful arrival of the packet. Alternative signaling to reduce overhead may be explored such as piggybacking the cancelling-information on other traffic. Clearly, the solution depends on the latency requirements and whether there is any communication from the receiver to the source. Furthermore, the efficiency of cancellations may be improved since the current solution mandates one signal per replicated packet. Lastly, as proposed in RAW [15], the necessity to utilize a mechanism such as RPE could be evaluated continuously by the forwarding plane.

Identifying and selecting paths to use with RPE clearly has impact on its performance. This subject was outside of our current scope yet requires investigation before a full implementation can be made. It should also include utilizing RPE over two paths with differing characteristics such as link quality and hop count.

The evaluation of RPE needs to be broadened to include more realistic propagation models and different traffic patterns, as well as varied topologies. These could include larger networks to observe scalability, and topologies from [18] for comparison with PAREO mechanisms. Experiments in real-world scenarios and testbeds would also be valuable.

Our implementation is limited to two disjoint paths, but extra paths could be implemented if the network has a denser topology. This would increase reliability further with a drawback of increased energy consumption.

In the current design, the traffic is always scheduled at Path A. However, implementing load balancing decreases energy consumption by distributing the load at both paths.

## Figures and Tables

**Table 1:** General parameters of the simulator.

Description	Value
Slotframes per run	21,000
Packet size	127 bytes
DAO ${}^{1}$ period	60 s
Scheduling function	MSF
Slot duration	10 ms
Slotframe length	101 slots
RPL ${}^{2}$ mode	Non-storing
Enhanced beacon probability	0.16
Clock max drift	30 ppm
Keep alive interval	10 s
Number of physical channels	16
Number of packets	2000
Packet interval	1 pkt/10 slotframes
Maximum retransmissions	4
Number of runs	30

${}^{1}$ Destination Advertisement Object (DAO). ${}^{2}$ Routing Protocol for Low-Power and Lossy Networks (RPL).

**Table 2:** Maximum and minimum theoretical latencies.

τ	Latency Minimum [s]	Latency Maximum [s]
0	0.04	16.16
1	0.04	16.17
8	0.04	16.24
816	0.04	24.32
1624	0.04	32.40
OP	0.07	16.28

**Table 3:** Max latencies for different link qualities.

PRR	Single Path [s]	τ=1 [s]	τ=8 [s]
70%	13.16	13.18	13.25
80%	12.16	10.14	11.23
90%	9.13	5.09	5.17

**Table 4:** The 99th percentile latencies for different link qualities.

PRR	Single Path [s]	τ=1 [s]	τ=8 [s]
70%	9.21	7.49	7.53
80%	7.08	4.89	4.96
90%	4.08	2.19	2.23

## Abbreviations

The following abbreviations are used in this manuscript:

ACK	Acknowledgement
BER	Bit Error Rate
DAG	Directed Acyclic Graph
DAO	Destination Advertisement Object
DetNet	Deterministic Network
IEEE	Institute of Electrical and Electronics Engineers
IETF	Internet Engineering Task Force
IoT	Internet of Things
IIoT	Industrial Internet of Things
IWSN	Industrial WSN
LFC	The Leapfrog Collaboration
PAREO	Packet Retransmission, Replication, Elimination, and Overhearing
PCE	Path Computation Element
PDR	Packet Delivery Ratio
PRR	Packet Reception Ratio
RAW	Reliable and Available Wireless
RPE	Reverse Packet Elimination
RPL	Routing Protocol for Low-Power and Lossy Networks
RX	Receiving
TSCH	Time-Slotted Channel Hopping
TX	Transmitting
WSN	Wireless Sensor Network
6TiSCH	IPv6 over the TSCH mode of IEEE 802.15.4e
